# Three-dimensional talar shape seems not a factor in chronic mechanical ankle instability

**DOI:** 10.1186/s12891-025-09458-2

**Published:** 2026-01-08

**Authors:** Markus Wenning, Lukas Klein, Daniel Heller, Thomas Lange, Hagen Schmal, Jan Kühle, Jörg Bayer

**Affiliations:** 1 OrthoTeam Freiburg, Orthopaedic Surgeons, Basler Str. 65, Freiburg, Germany; 2 BDH Klinik Waldkirch – Orthopedic Surgery, Waldkirch, Germany; 3 Department of Orthopaedic and Trauma Surgery, University Medical Center, Faculty of Medicine, Freiburg im Breisgau, Germany; 4 Center for Diagnostic and Therapeutic Radiology, Medical Physics, University Medical Center, Faculty of Medicine, Freiburg im Breisgau, Germany; 5 Department of Orthopaedic Surgery, Odense University Hospital, Odense, Denmark; 6 Department of Orthopaedic and Trauma Surgery, Schwarzwald-Baar Klinikum, Villingen-Schwenningen, Germany

**Keywords:** Chronic ankle instability, Talar shape, Mechanical ankle instability, 3D-MRI

## Abstract

**Background:**

There is a long-lasting discussion on whether the anatomical shape of the talus is a predisposing factor in the development of chronic ankle instability (CAI). The progression from two- to three-dimensional imaging techniques allows for a new investigation on this topic.

**Methods:**

MRI studies of 25 young and healthy adults with CAI and 25 controls without CAI were conducted in neutral-null position and in plantarflexion-supination. The talar angle and the talar radius were transposed to a three-dimensional approach and compared between the groups and the positions.

**Results:**

There was no significant difference in the talar angle nor the talar radius between the groups. Plantarflexion-Supination did not lead to a significant change in tibiotalar configuration associated with the two parameters.

**Conclusion:**

Three-dimensional talar shape is not significantly different between patients suffering from CAI and healthy controls. This supports the interpretation that the dynamic congruency of the joint, which is influenced by ligamentous integrity remains the main anatomical component in mechanical ankle instability.

**Trial registration:**

The study protocol was prospectively registered at the German Clinical Trials Register (#DRKS00016356) on 05/11/2019.

## Background

Chronic ankle instability (CAI) is a common phenomenon in athletic patients following an acute lateral ankle sprain [1]. The risk of developing chronic ankle instability following an acute sprain has been reported to be as high as 20–40% [1]. In 2013 the international ankle consortium defined CAI by the presence of recurrent sprains, pain, swelling and feelings of “giving way” [2]. Since then, research focusing on chronic ankle instability offered new insights on the pathogenesis and potential risk factors. Consequently, previous assumptions on this common injury have been re-evaluated [3]. Furthermore, current perception of CAI includes the two intertwining etiologies of mechanical ankle instability (MAI) and functional ankle instability (FAI) [4]. Present research is in the midst of separating these two etiologies and clarifying the influence of each type of impairment on the clinical appearance of CAI [4, 5].

Functional impairments include deficits in postural control and neuromuscular activation, while mechanical deficits consist of increased anterior drawer and talar tilt test, leading to a significant loss in osseous talar containment [5, 6].

Previous research applying two-dimensional radiological analysis suggested that the anatomical shape of the talus might be significantly different between healthy controls and patients suffering from CAI: For example, Frigg et al. described the radial shape of the talar surface as a factor, where a smaller radius and a greater distance from the talus’ center were factors determining CAI [7, 8].

The talar height, representing the distance between the center of the talus and the tibiotalar articular surface or overlap, was also described as a potential factor which might increase the tilting angle and consecutively alternate force vectors in such a manner that an uneven impact would lead to more rapid angular velocity during supination [8, 9]. Additionally, the medial and lateral osseous containment of the talus in plain radiographs has also been described to be significantly different between patients with CAI and healthy controls. In summary, healthy ankles maintain their medial and lateral malleolar overlap even in end-range plantarflexion, leading to an increased medio-lateral stability [10]. This finding was supported by other studies, comparing the tibial and fibular distal tips to the talar center-of-rotation [8].

Due to the progression of imaging modalities, these factors have been partially transferred into multi-slice imaging such as MRI and CT scans. While it has become clear, that simple two dimensional image analysis is insufficient for diagnosing MAI correctly, there have been recent studies focusing on 3-dimensional articular configuration [11–14]. For example, it has been shown that the reduction of three-dimensional articular fibulotalar surface contact during plantarflexion-supination is a good indicator for mechanical ankle instability [6]. From a clinical perspective, it has also become apparent that the rotational, three-dimensional component in ankle instability might be a decisive factor defining the risk of re-injury and chronification [15, 16]. Therefore, the reconstruction or reinsertion of injured ligaments should evaluate the anatomical situation and the consecutive predisposition for ankle instability in each patient [17]. Thus, it is important to re-evaluate the former findings from two-dimensional imaging using three-dimensional imagery to further increase the understanding of mechanical contributions leading to CAI.

The aim of this study was to critically appraise the available factors of talar shape in CAI originating from two-dimensional radiographs in an effort to transfer them into a three-dimensional analysis. Under consideration of the aforementioned literature, we hypothesized that the talar radius and the angle of the tibiotalar overlap would be decreased in MAI compared to healthy controls with a special focus on the medio-lateral distribution across the talus and the corresponding behavior during plantarflexion-supination.

## Methods

This study was approved by the local ethics committee EKFR #118/19 (Ethikkommission Freiburg, Germany) and it was conducted according to the Declaration of Helsinki in its current form. All participants declared written informed consent prior to participation. This is a follow up analysis of a previously published cohort [6], focusing on the aforementioned aspects.

### Participants

We recruited two groups of *n* = 25 each from a random community sample of students: (A) suffered from mechanical ankle instability, which was defined according to the recommendations of the international ankle consortium [2] with a history of at least one severe lateral ankle sprain, followed by recurrent sprains, feelings of giving way, pain and/or swelling. Inclusion criteria further required a Cumberland Ankle Instability Tool score < 24, presence of mechanical ankle instability upon clinical examination (talar tilt and anterior drawer testing) and athletic activity of > 5 h/week. (B) healthy controls were defined as people with no symptoms of ankle instability, no history of a previous sprain and no clinical manifestation of ankle instability.

Exclusion criteria were a history of surgery around the ankle, a recent sprain < 3 months prior to participation, generalized hyperlaxity or indecisive findings during clinical examination.

### MRI

All MRI experiments were performed on a Magnetom Trio 3 T system (Siemens Healthineers, Erlangen, Germany), using an 8-channel multipurpose coil (NORAS MRI Products, Germany) for signal reception. The protocol consisted of a 3D turbo-spin echo (TSE) sequence with GRAPPA parallel imaging acceleration by a factor of 2. The 3D imaging volume consisted of 128 sagittal slices with an in-plane resolution of 0.5 mm and a slice thickness of 0.6 mm. The MRI was acquired in neutral position with the foot firmly attached to the previously described [12], custom made device and in a plantarflexed and supinated position, of which the extent of the joint’s position could be read from.

### Postprocessing and primary outcome

For all of the postprocessing of the MRI data, a browser-based framework for medical image analysis (Nora Medical Imaging Platform, Freiburg, Germany) was used [18].

**Fig. 1:**
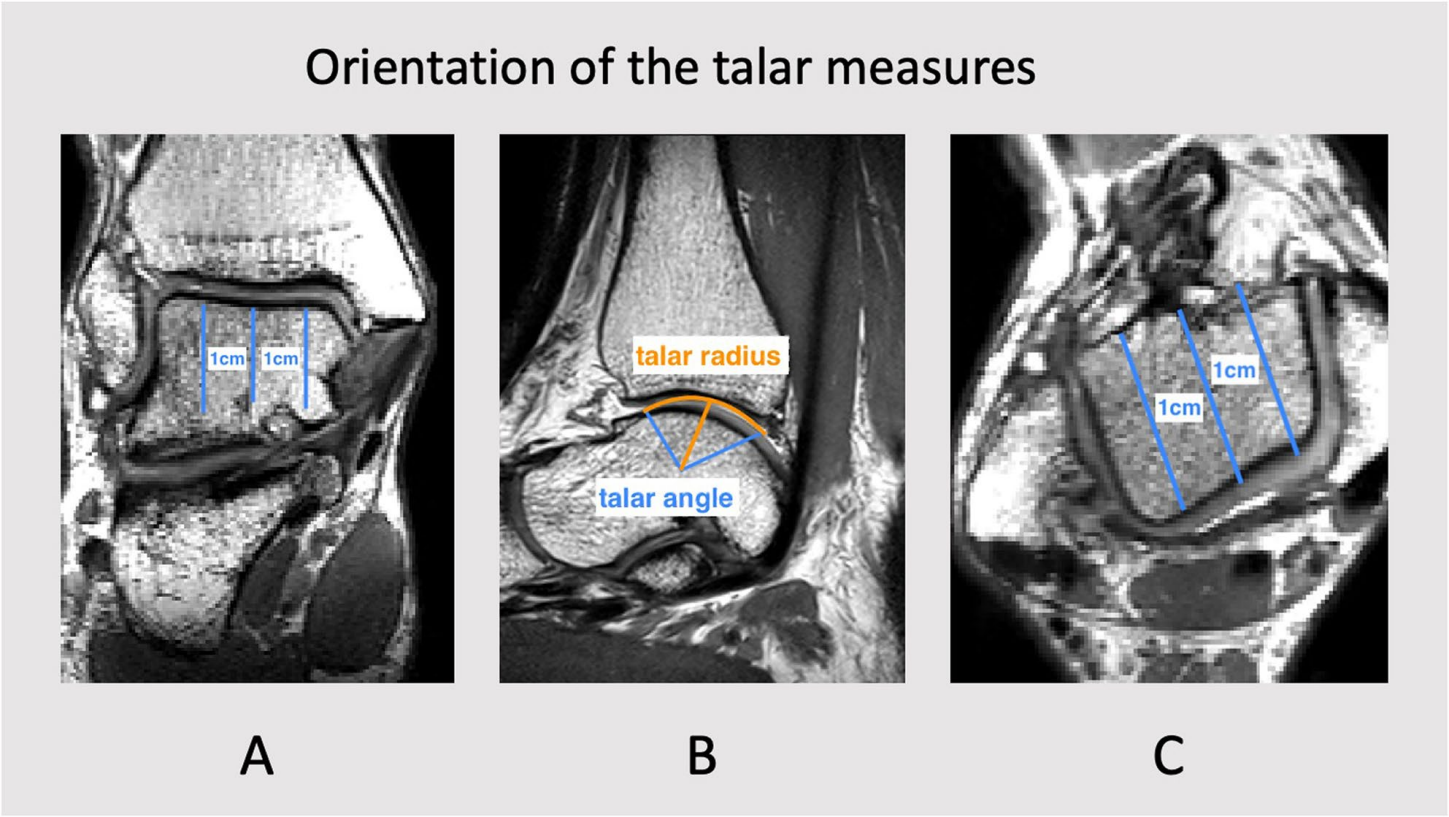
Display of the orientation of the talar measures in an excerpt of a 3D SPACE MRI. **A** coronar view, **B **sagittal view, **C **transversal view

Primary outcomes of the present study were the talar radius and the angle of tibiotalar coverage (talar angle, see Fig. 1). These factors were deducted from previously published literature on 2-dimensional radiographic studies as mentioned above [7, 8] and were consecutively applied to three-dimensional measures.

The central starting point was defined according to the longitudinal axis of the distal tibia in the sagittal as well as the coronal plane defined by two separate measurements along the tibial anatomical axis. It has been described that this will approximate the perpendicular of the upper ankle joint as a mechanical correlate of the application of the body weight [19–21]. The sagittal alignment was performed parallel to the lateral talar edge at the height of the talar center. The center of the talar radius was defined as midway between the cartilage surfaces of the upper and lower ankle joints following the central anatomical axis of the tibia in the three-dimensional projection as described before. The talar angle was defined as the angle built by two straight lines from the talar center to the anterior and posterior edges of tibiotalar cartilage contact. The talar radius was defined as the best-fit to the curvature of the talar dome in the area of tibiotalar cartilage contact (Fig. 1B).

Additionally, these measures were taken 1 cm medial and 1 cm lateral to the primary talar center (Fig. 1A and C) for the medio-lateral distribution to deduct a stepwise estimation of the shape of the talar trochlea.

### Statistical analysis

Statistical analysis was conducted using the Statistical Package for the Social Sciences (SPSS) Version 27 (IBM Corp., Armonk, NY, USA). Graphical display was performed using Veusz (v. 3.0.1 by Jeremy Sanders).

For statistical comparison, after checking for normal distribution using the Shapiro-Wilk test, a single-factor ANOVA was carried out with the two-level factor group (MAI vs. CON). The level of significance was set at *p* < 0.05. Effect sizes were interpreted following Cohen (small: 0.01, medium: 0.06, and large: 0.12.).

## Results

Table 1 presents the results of the talar angle and Table 2 shows the results of the talar radius. There were no significant differences between the groups. In both groups the talar angle decreased from the center of the talus to its borders which was consistent in both positions of the ankle. There were no significant differences in any values.

**Table 1:** Talar angle as a measure of tibiotalar containment in neutral-null and plantarflexion-supination in degrees (±SD)

Neutral-Null	Lateral	Central	Medial
CON	43.1 (±12.8)	63.6 (±6.6)	46.9 (±9.0)
MAI	41.2 (±12.2)	64.0 (±7.3)	47.8 (±11.1)
Sign.	n.s.	n.s.	n.s.
Plantarflexion-Supination	Lateral	Central	Medial
CON	39.9 (±16.7)	62.7 (±12.4)	49.5 (±8.9)
MAI	36.1 (±17.9)	62.9 (±8.8)	47.8 (±12.7)
Sign.	n.s.	n.s.	n.s.

**Table 2:** Talar radius in neutral-null and plantarflexion-supination in mm (±SD)

Neutral-Null	Lateral	Central	Medial
CON	19.7 (±1.6)	20.2 (±1.0)	20.4 (±1.6)
MAI	19.6 (±2.9)	20.3 (±1.8)	20.4 (±2.7)
Sign.	n.s.	n.s.	n.s.
Plantarflexion-Supination	Lateral	Central	Medial
CON	18.5 (±2.5)	20.2 (±1.0)	19.5 (±1.6)
MAI	18.2 (±2.3)	20.1 (±1.6)	19.7 (±2.0)
Sign.	n.s.	n.s.	n.s.

The talar radius decreased in both groups from medial to lateral in a comparable manner from 20.4 mm medially to 19.6 vs. 19.7 mm laterally. Also, there were no significant differences between the groups.

## Discussion

In this cohort study with a well-defined group of patients suffering from mechanical instability (MAI) and a healthy control group (CON) we replicated historical measures originating from 2D roentgenology and transferred them into a 3D MRI setting. We had to rely on historical 2D plain radiographs since the patients in our study group were inconsistently diagnosed with this examination, thus a thorough measurement o 2D values was not applicable in this cohort. We assessed whether the osseous shape of the talus in the segment of the tibiotalar joint had a significant difference between MAI and CON. Our data from three-dimensional MRI show no evidence that the osseous shape of the talus may have a relevant impact on the development of chronic mechanical instability. It had been postulated previously, that certain differences may be present in chronic ankle instability [7, 8, 10], a finding, which should be reconsidered. Even more so, because there was no significant difference in a plantarflexed position either. Due to the conus-like shape of the talar surface, it is prone to an excessive joint excursion in plantarflexion. However, the data in this study suggests that the osseous configuration has no additional effect to this potential incongruency of the talocrural joint.

The potential increase in specificity of the osseous talocrural pathology by applying 3D-measurements, derived from the interpretation that a very small talar radius may further reduce the tibiotalar contact area and secondly the anatomical containment of the joint. Moreover, it was previously expected that the medial and/or lateral talar shoulder would have had a significantly larger radius in healthy ankles, because this would then increase the tibiotalar congruency and form fit of the joint, reducing inherent joint play. Since the 2-dimensional analysis of the talus could fall short in analyzing these specific subtypes, a 3D analysis seemed to be more apt. Nevertheless, from the results of this study, this hypothesis could not be proven.

This statement of our research has to be discussed according to our measuring algorithm. Our measuring technique seems not very common, but to the best of our knowledge, this is the first time that the osseous shape of the talus has been analyzed in 3D-MRI. The international biomechanical society has not yet defined specific means of the “center-of-talus”, which is why we proposed to use the method applied in this study. It is a challenge to properly define the center of the talus due to its inhomogenous shape. In our approach we chose the central axis of the tibia and then mid-way between upper- and lower ankle joint as the most appropriate landmarks to address our study hypothesis. From our biomechanical standpoint, this should be the point where the major impact of the body’s weight will effectuate and thus result in or enhance a potential instability.

While the three-dimensional osseous shape of the talus has been subject to research in other fields such as cartilage lesions, there are few analyses focusing on the interaction between talar shape and lateral ankle instability [22, 23]. In 2010 Bonnel et al. reported on a detailed morphological analysis of the talus and a possible correlation with ankle instability [24]. Three different types of talar shape were identified and an increased risk of lateral ankle instability, due to a rather narrow shape of the posterior part of the talus, was postulated. This narrow posterior talus may decrease the osseous containment during plantarflexion and therefore facilitate lateral instability [24]. In contrast to this finding, our current results from 3D-MRI do not show a difference between CON and MAI attributed to the radius of the talus. Therefore, it underlines that the primary pathomechanism in lateral ankle instability is a ligamentous insufficiency and not merely an anatomical predisposition due to the shape of the talus. Thus, the dynamic loss of talocrural containment represented e.g. by cartilage contact area seems a more apt estimation of the mechanical contribution to lateral ankle instability [6, 12]. From a clinical perspective this is important since the repair of periarticular soft tissue remains the treatment of choice in CAI, while addressing the osseous shape of the talus by surgery does not seem feasible.

## Data Availability

Data is provided within the manuscript. Further data can be made available upon reasonable request.
